# The Diagnosis and Treatment of Syphilis

**Published:** 1887-06

**Authors:** 


*The Diagnosis and Treatment of Syphilis.* By TOM ROBINSON, M.D. Pp. 138. London: J. & A. CHURCHILL. 1886.

Dr. Tom Robinson has, he tells us, been indebted largely to private practice for bringing him into "contact with the drama of syphilis." "Dramatic" might in some degree be said to characterise this book. The language, at least, is neither prosaic nor tame. For instance: "We now pass on to the last of my venereal sores. I am always unwilling to abolish old names; but . . . we are apt to be fascinated by the division of syphilis into primary, secondary, and tertiary syphilis. A patient of mine came to me one day saying he had *fourthlies.* Such divisions are unscientific, and certainly not natural. I am acquainted with the other divisions which have been used by Virchow and others." We trust that, on the other side, Virchow has, as a matter of fairness, had an opportunity of studying the list of what Dr. Tom Robinson calls "his venereal sores."

"The long bones are most commonly influenced by the necrosial process; whilst the flat bones become carial." If the author, though "unwilling to abolish old names," is occasionally driven to use new ones, we must surely admit that the novelties sound musical. "Necrosial" and "carial" vaguely suggest something which gods are supposed to eat and drink.

"Iodide of Potassium.—This drug will eat up all syphilitic products with marvellous precision." We have thought that, if a fiery dragon happened to be found in a travelling caravan of horrors, this sentence would be an excellent label to hang over it.

Such is the book. Not improperly might it be named, in the author's own words, "The Drama of Syphilis."

